# Wound Healing Effects of Polyethylene Glycol Loxenatide via PI3K/AKT Pathway: In Vitro Evaluation in Human Keratinocytes

**DOI:** 10.1155/jdr/6683809

**Published:** 2026-04-22

**Authors:** Zhiyi Zhao, Han Yue, Weijie Xu, Song Gong, Shiying Shao

**Affiliations:** ^1^ Division of Endocrinology, Department of Internal Medicine, Tongji Hospital, Tongji Medical College, Huazhong University of Science and Technology, Wuhan, China, hust.edu.cn; ^2^ Hubei Clinical Medical Research Center for Endocrinology and Metabolic Diseases, Wuhan, Hubei, China; ^3^ Branch of National Clinical Research Center for Metabolic Diseases, Wuhan, Hubei, China

**Keywords:** glucagon-like peptide-1, keratinocyte, loxenatide, wound healing

## Abstract

**Aim:**

Glucagon‐like peptide‐1 receptor agonists (GLP‐1RAs) hold clinical promise in promoting wound healing. This study investigated the pro‐healing effect and potential molecular mechanism of polyethylene glycol loxenatide (PEG‐Lox).

**Materials and Methods:**

HaCaT cells were incubated in high glucose (HG) condition with or without PEG‐Lox. Cell proliferation and viability were assessed by Cell Counting Kit 8 (CCK8) assay and PI staining. Cell migration was evaluated via Transwell and scratch wound healing assays. Transcriptomic analysis was conducted to identify the potential differentially expressed genes (DEGs). Western blotting was performed to validate protein expression levels of identified DEGs.

**Results:**

Cell proliferation and viability were significantly improved in the PEG‐Lox‐treated group when compared with the control group. Transwell assay showed that HG inhibited the migration of HaCaT cells, whereas PEG‐Lox treatment markedly resulted in a 1.7‐fold increase in the number of migrating cells (*p* < 0.0001). The scratch closure rate was 32.83% after PEG‐Lox intervention, which was higher than that in the control group (24.42%, *p* < 0.001). Transcriptomic analysis found early growth response factor 1 (EGR1) to be the key DEG under PEG‐Lox treatment. The inhibition of PI3K/AKT signaling pathway by LY294002 down‐regulated the expression of EGR1 and impaired the proliferation and migration of HaCaT cells.

**Conclusions:**

Our study suggested that GLP‐1RA PEG‐Lox could promote wound healing by activating the PI3K/AKT/EGR1 signaling pathway, which further uncovered the potential pro‐healing effect of GLP‐1RAs in wounds.

## 1. Introduction

Glucagon‐like peptide‐1 (GLP‐1), an incretin hormone secreted by gut enteroendocrine cells, plays an essential role in maintaining glucose homeostasis primarily by stimulating insulin secretion and inhibiting glucagon release [1]. GLP‐1 receptor agonists (GLP‐1RAs) have gained prominence in the treatment of type 2 diabetes mellitus (T2DM) due to their multiple clinical beneficial effects, including glucose control, cardiovascular protection, and weight reduction [2].

Chronic wounds are becoming increasingly prevalent among patients with T2DM, frequently resulting in severe complications such as amputation and placing a significant burden on healthcare systems [3]. Beyond the established metabolic effects, preclinical studies have recently revealed pleiotropic roles of GLP‐1RAs in wound repair by activating GLP‐1 receptor (GLP‐1R) on various cell types, including vascular endothelial cells [4], keratinocytes [5], fibroblasts [6], and immune cells [7, 8]. Aronis et al. [9] demonstrated in an in vitro study that the GLP‐1RA exendin‐4 can enhance angiogenesis in human umbilical vein endothelial cells (HUVECs) through serine/threonine kinase (AKT), Protein Kinase C (PKC) and Src pathways. A similar study reported that liraglutide could enhance the function of HUVECs in the high glucose (HG) condition by activating the AMP‐activated kinase (AMPK) signal [10]. In parallel, research on diabetic rat models demonstrated that the exendin‐4 directly improved fibroblast function by regulating matrix metalloproteinase‐9 (MMP‐9)/tissue inhibitor of metalloproteinase‐1 (TIMP‐1) ratio [11]. In addition to vascular endothelial cells and fibroblasts, an in vivo study reported that exenatide significantly increased the number of circulating endothelial progenitor cells (CD34/KDR) in the wounds of diabetic rats, as well as enhanced capillary density [12].

Accumulating evidence indicates that the migration and proliferation of keratinocytes are critical throughout nearly all stages of wound healing [13, 14]. In diabetic wounds, chronic hyperglycemia disrupts re‐epithelialization by impairing keratinocyte differentiation and function [15–17]. Despite their established role to healing, only two studies have investigated the effects of GLP‐1RAs on keratinocytes. One study by Zhang et al. [18] demonstrated that GLP‐1RA liraglutide promoted wound re‐epithelialization and enhanced the proliferation and migration of HaCaT cells under normal glucose (NG) condition by regulating the Myo1c/Dock5 pathway. This study did not further comprehensively assess the effects of GLP‐1RA on HaCaT cell function under HG condition in vitro [18]. Another study conducted by Nagae et al. [5] reported that liraglutide promoted HaCaT cell proliferation and wound healing potentially via phosphatidylinositol 3 kinase (PI3K)/AKT signaling pathway. However, the downstream mechanisms remain unexplored, highlighting a significant gap in understanding the complete signaling cascade. Therefore, further investigation is needed to elucidate the downstream effector molecules and precise regulatory mechanisms involved, which could provide critical insights into the therapeutic potential of GLP‐1RAs in diabetic wound healing.

Polyethylene glycol loxenatide (PEG‐Lox) is an ultra‐long‐acting GLP‐1RA with its prolonged half‐life conferred by polyethylene glycol (PEG) modification [19]. Although multiple studies have evaluated its benefits on glucose control and body mass index (BMI) [20], the effects of once‐weekly PEG‐Lox in diabetic wound healing and its potential mechanisms have not yet been investigated. Therefore, this study aims to investigate the pro‐healing effect and potential molecular mechanisms of ultra‐long‐acting GLP‐1RA PEG‐Lox in HaCaT cells under HG condition, addressing the existing gaps with a specific focus on elucidating the downstream effector molecules and precise regulatory mechanisms within the PI3K/AKT signaling pathway.

## 2. Materials and Methods

### 2.1. Cell Culture and Treatment

Human immortalized keratinocyte HaCaT cells were purchased from Servicebio (Wuhan, China), authenticated by short tandem repeat (STR) profiling, and confirmed to be mycoplasma‐free. The cells were cultured in Dulbecco’s modified Eagle’s medium (DMEM) supplemented with 10% fetal bovine serum (FBS, Gibco) and 1% penicillin‐streptomycin at 37°C in 5% CO_2_ atmosphere. At ~80% confluence, cells were treated with 100 nM PEG‐Lox (Hansoh Pharma, China) and/or 30 mM HG for 24 h or 48 h. In certain groups, cells were pre‐treated with 10 μM PI3K inhibitor LY294002 (Beyotime, China) for 2 h prior to PEG‐Lox and/or glucose exposure according to previous studies [21–23]. The control group was maintained under 2.8 mM NG condition.

### 2.2. Cell Proliferation and Viability Assay

Cell proliferation was assessed using the Cell Counting Kit 8 (CCK8) assay (Dojindo, Japan). Briefly, HaCaT cells were seeded at a density of 1 × 10^3^ cells per well in 96‐well microtiter plates and treated according to the experimental groups for 24 or 48 h. Subsequently, the medium was replaced with fresh medium containing 10 μL of CCK‐8 reagent. The plates were then incubated at 37°C in a humidified atmosphere containing 5% CO_2_ for 1 h. The optical density (OD) of the supernatant was measured at a wavelength of 450 nm using a microplate reader (BioTek, USA).

Cell viability was assessed using Calcein AM/propidium iodide (PI) staining (Dojindo, Japan). HaCaT cells were seeded into a 24‐well plate and treated with PEG‐Lox/glucose for 24 h. The cells were then washed with PBS and incubated with Calcein AM (2 µM) for 30 min at room temperature. Subsequently, the dye was replaced with PI (3.5 µM) and cells were incubated at room temperature for 5 min. Live and dead cells were then observed under an inverted fluorescence microscope (Olympus, Japan), and images were captured. The percentage of live/dead cells was quantified using ImageJ software.

### 2.3. Cell Scratch Assay

The in vitro scratch wound healing assay was performed as previously described [24]. Briefly, HaCaT cells were seeded into 6‐well plates at a density of 5 × 10^5^ cells per well, and cultured to 90% confluence. A sterile 200 μL pipette tip was used to generate three parallel linear scratches per well, oriented perpendicular to plate markings for consistent imaging. Subsequently, the cells were cultured in serum‐free medium supplemented with experimental treatments. The width of each scratch was observed and photographed with an optical microscope at 0, 12, and 24 h. The scratch area was quantified using ImageJ software.

### 2.4. Cell Migration Assay

The cell migration was measured in 24‐well plates of Transwell filter chamber [25]. HaCaT cells were seeded into the upper chamber at a density of 5 × 10^4^ cells per well containing 200 µL serum‐free medium, and 600 µL medium containing 20% fetal bovine serum was added to the lower chamber. After incubation at 37°C and 5% CO_2_ for 24 h, migrated cells were fixed with 4% paraformaldehyde for 30 min and stained with 0.1% crystal violet. The number of cells was counted under the light microscope (Olympus, Japan).

### 2.5. Cell Cycle Assay

After 24 h of cell treatment, the cell cycle of HaCaT cells was analyzed using a cell cycle detection kit (Keygen, China). HaCaT cells were seeded in 6‐well plates at a density of 1 × 10^6^ cells per well and then collected after the interventions. Subsequently, cells were fixed overnight with 70% cold ethanol at 4°C, followed by washing with pre‑chilled PBS and staining with PI. The DNA content of HaCaT cells was detected by flow cytometry (Beckman, USA). The percentages of cells in the G0/G1, S, and G2/M phases were analyzed using Flowjo software (version 10.8.1).

### 2.6. RNA Sequencing and Bioinformatics

RNA isolation was performed using TRIzol reagent (Invitrogen, USA). cDNA libraries were prepared using the Illumina‐compatible total RNA‐seq library preparation kit (Vazyme, China). Sequencing was executed on the Illumina NovaSeq 6000 platform (Vazyme, China). Differentially expressed genes (DEGs) between groups were determined via the DESeq2 R package (v1.20.0), applying thresholds of an adjusted *p*‐value <0.05 and an absolute fold change ≥1.5. Functional enrichment assessments for DEGs, focusing on Gene Ontology (GO) terms and Kyoto Encyclopedia of Genes and Genomes (KEGG) annotations, were carried out through the Metascape platform (https://metascape.org).

### 2.7. Western Blot Analysis

Total protein was extracted using RIPA lysis buffer. Protein quantification was performed using a BCA protein quantification kit (Beyotime, China). The extracted proteins were separated via sodium dodecyl sulfate‐polyacrylamide gel electrophoresis (SDS‐PAGE) and subsequently transferred to a polyvinylidene fluoride (PVDF) membrane. Membranes were blocked with 5% non‑fat milk in TBST and then incubated overnight at 4°C with primary antibodies PI3K (RRID: AB_2714032, Abcam, UK), phosphorylated PI3K (p‐PI3K) (RRID: AB_3024789, Abcam, UK), AKT (RRID: AB_11156777, Abcam, UK), phosphorylated AKT (p‐AKT) (RRID: AB_722678, Abcam, UK), early growth response factor‐1 (EGR1) (RRID: AB_2715513, Abcam, UK), Cyclin D1 (RRID: AB_302478, Abcam, UK), Cyclin D3 (RRID: AB_2215410, Abcam, UK), and β‐actin (RRID: AB_955200, Abcam, UK). Following this, the membrane was co‐incubated with goat anti‐rabbit IgG secondary antibody (Abcam, UK) at room temperature for 2 h. The proteins of the membranes were visualized with chemiluminescent HRP substrate (Beyotime, China).

### 2.8. Statistical Analysis

Quantitative data from three independent biological replicates were presented as mean values ± standard deviation. Intergroup comparisons employed two‐tailed unpaired Student’s *t*‐test, while multi‐group comparisons utilized One‐way or two‐way analysis of variance (ANOVA) with Tukey’s post hoc analysis. Statistical computations were conducted in GraphPad Prism (v9.5), with *p* < 0.05 defining statistical significance.

## 3. Result

### 3.1. PEG‐Lox Promoted the Viability and Proliferation of HaCaT Cells

To evaluate the effect of PEG‐Lox on HaCaT cell proliferation, a CCK‐8 assay was performed. In the preliminary experiment, no significant difference in OD values was observed between the 50 nM PEG‐Lox‐treated group and the control group (data not shown). In contrast, treatment with 100 nM PEG‐Lox for 24 h not only promoted cell proliferation under NG condition (OD value in Con group, 0.95 ± 0.02 vs. Lox group, 1.2 ± 0.05, *p* < 0.0001) but also reversed the impaired proliferation under HG condition (OD value in HG group, 0.82 ± 0.02 vs. HG + Lox group, 0.99 ± 0.04, *p* < 0.001, Figure 1A). This effect was maintained at the 48 h time point, with PEG‐Lox treatment resulting in higher OD values in both NG (1.40 ± 0.03 vs. 1.61 ± 0.05, *p* < 0.0001) and HG conditions (1.17 ± 0.02 vs. 1.27 ± 0.01, *p* < 0.01, Figure 1A). Additionally, Calcein AM/PI staining was performed in HaCaT cells to evaluate the effect of PEG‐Lox on cell viability. Compared to the control group, the proportion of live cells sharply decreased after 30 mM glucose incubation, which could be significantly mitigated by PEG‐Lox intervention (*p* < 0.001, Figure 1B, C). Furthermore, cell cycle analysis identified that PEG‐Lox treatment significantly increased the proportion of cells entering the S phase in both NG and HG conditions, representing 1.2‐ and 1.3‐fold increases, respectively (*p* < 0.01, Figure 1D, E). Western blot analysis further indicated that PEG‐Lox treatment caused an elevation in the expression of the proliferation‐related proteins cyclin D1 and cyclin D3 (rising rate of Cyclin D1:41% in NG condition, 40% in HG condition, *p* < 0.01; rising rate of Cyclin D3:33% in NG condition, 31% in HG condition, *p* < 0.01, Figure 1F). These findings suggest that PEG‐Lox can promote the viability and proliferation of HaCaT cells under both NG and HG conditions.

Figure 1PEG‐Lox promoted the viability and proliferation of HaCaT cells. (A) CCK8 assay for the cell proliferation evaluation. (B) Calcein AM/PI staining for the assessment of HaCaT cell viability. Green represents living cells; Red represents dead cells. Scale bar: 100 µm. (C) Quantitative analysis of the proportion of living cells. (D) Representative images of flow cytometry for the cell cycle distribution of the HaCaT cells. (E) Quantitative analysis of the cell cycle distribution according to flow cytometry. (F) Western blotting analysis of Cyclin D1 and Cyclin D3. (G) Quantitative analysis of the protein level of Cyclin D1 normalized to β‐actin. (H) Quantitative analysis of the protein level of Cyclin D3 normalized to β‐actin. Data are presented as mean ± SD from three independent experiments. Statistical differences were evaluated by two tail unpaired Student’s *t* test and one‐way or two‐way ANOVA.  ^∗^ *p* < 0.05,  ^∗∗^ *p* < 0.01,  ^∗∗∗^ *p* < 0.001,  ^∗∗∗∗^ *p* < 0.0001. Con, control; HG, high glucose; Lox, PEG‐Lox.

**Figure:**
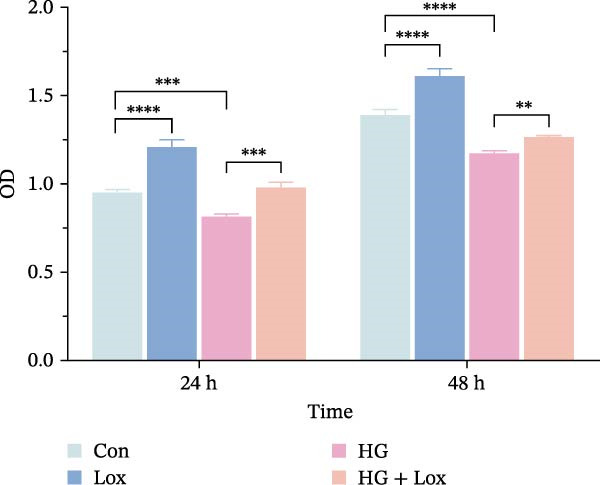
(A)

**Figure:**
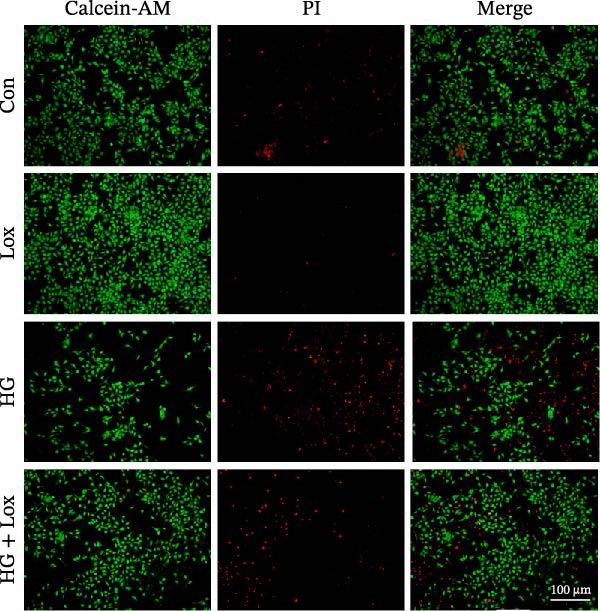
(B)

**Figure:**
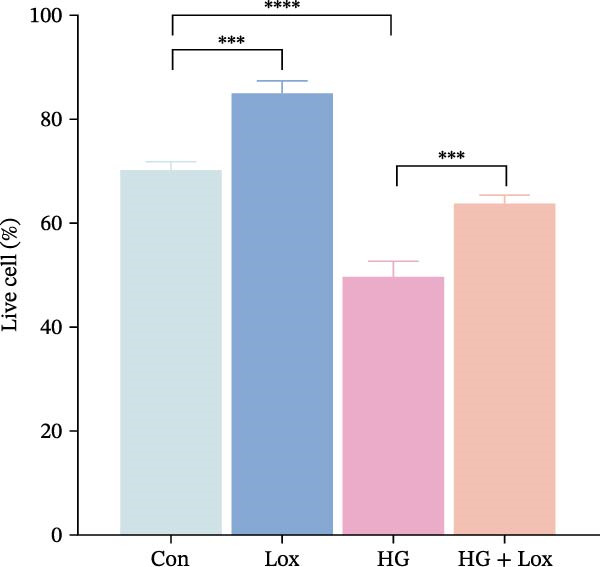
(C)

**Figure:**
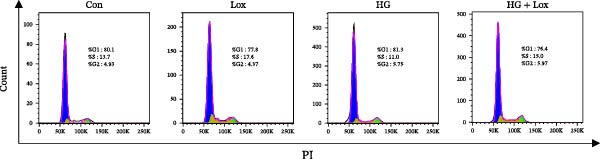
(D)

**Figure:**
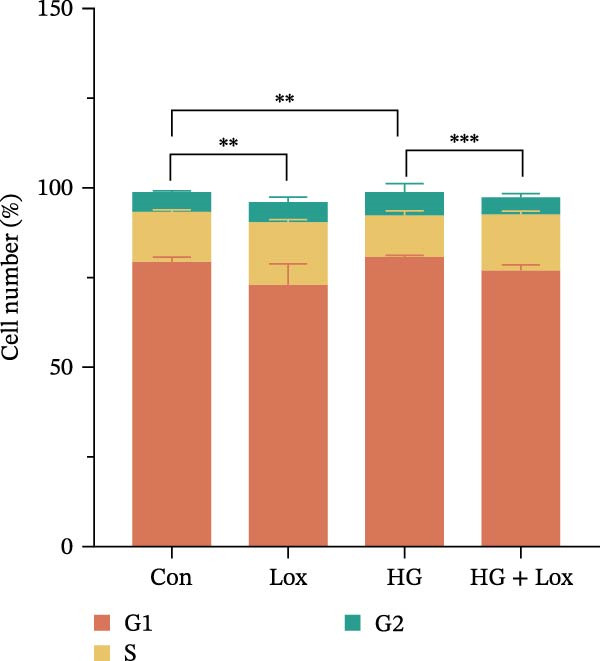
(E)

**Figure:**
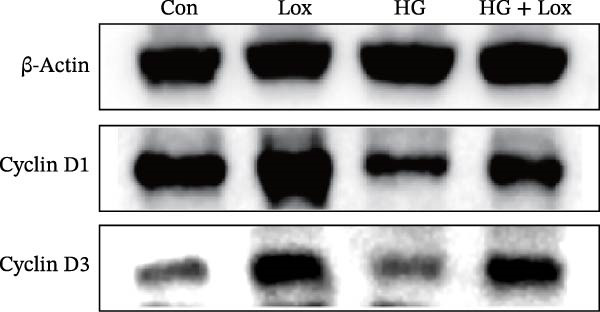
(F)

**Figure:**
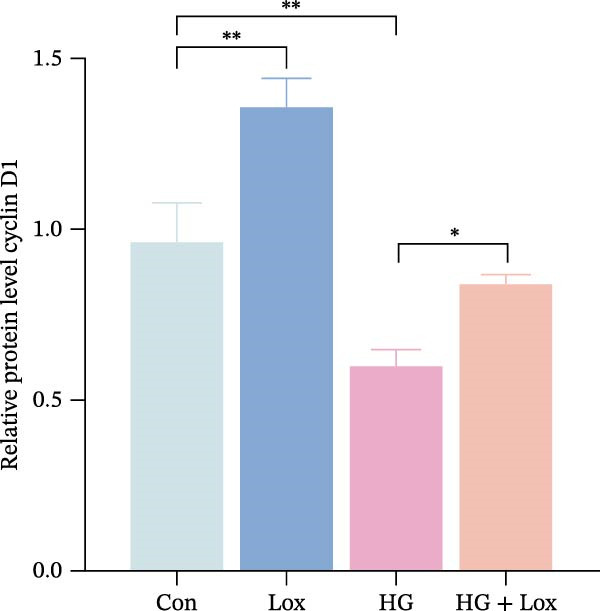
(G)

**Figure:**
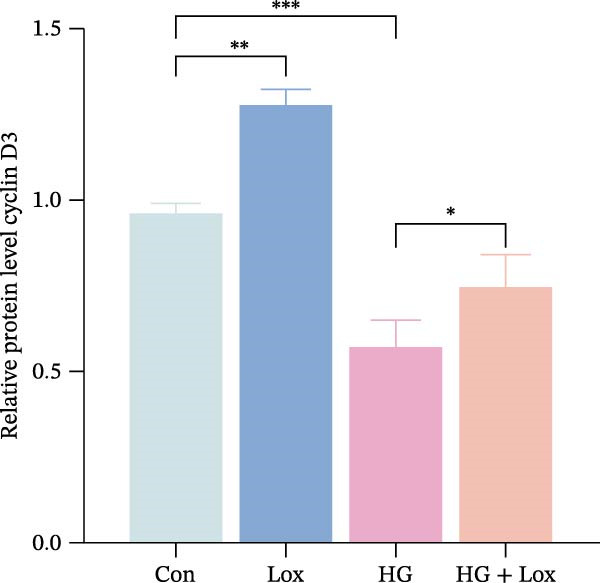
(H)

### 3.2. PEG‐Lox Promoted the Cell Migration In Vitro

To investigate the effect of PEG‐Lox on wound healing in vitro, a scratch assay was performed in HaCaT cells. As shown in Figure 2, HG condition significantly inhibited HaCaT cell migration compared with the control group (*p* < 0.01). Conversely, treatment with PEG‐Lox resulted in a substantial increase in the wound closure rate at 24 h post‐intervention (rising rate: 50% in NG vs. 26% in HG condition, *p* < 0.001, Figure 2A, B). Furthermore, results from the Transwell assay also demonstrated that HG inhibited the migration of HaCaT cells, whereas PEG‐Lox markedly increased the number of cell migration to 1.7‐fold under NG condition and to 1.3‐fold under HG condition (*p* < 0.0001, Figure 2C, D).

Figure 2PEG‐Lox promoted the cell migration in vitro. (A) Representative images of in vitro scratch assay captured at 12 hr and 24 h. The black dotted line indicates the scratch area. Scale bar: 200 µm. (B) Quantitative analysis of the wound area (%) in vitro. (C) Representative images of Transwell assay to evaluate cell migration. Scale bar: 100 µm. (D) Quantitative analysis of cell migration numbers of HaCaT cells. Data are presented as mean ± SD from three independent experiments. Statistical differences are evaluated by two tail unpaired Student’s *t* test and one‐way or two‐way ANOVA.  ^∗∗^ *p* < 0.01,  ^∗∗∗^ *p* < 0.001,  ^∗∗∗∗^ *p* < 0.0001. Con, control; HG, high glucose; Lox, PEG‐Lox.

**Figure:**
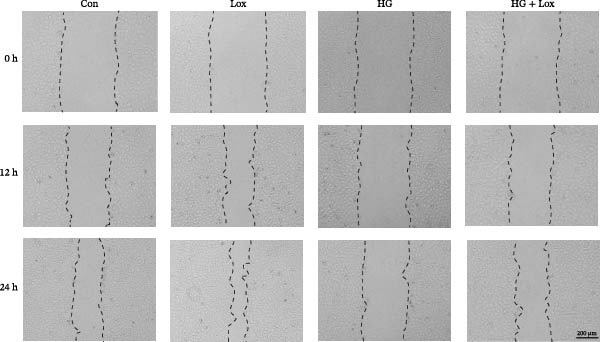
(A)

**Figure:**
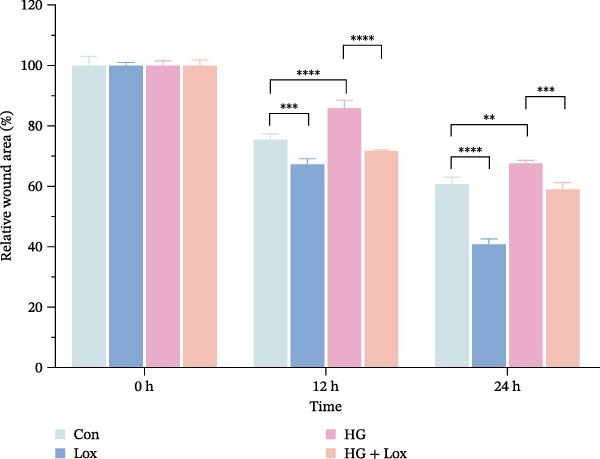
(B)

**Figure:**
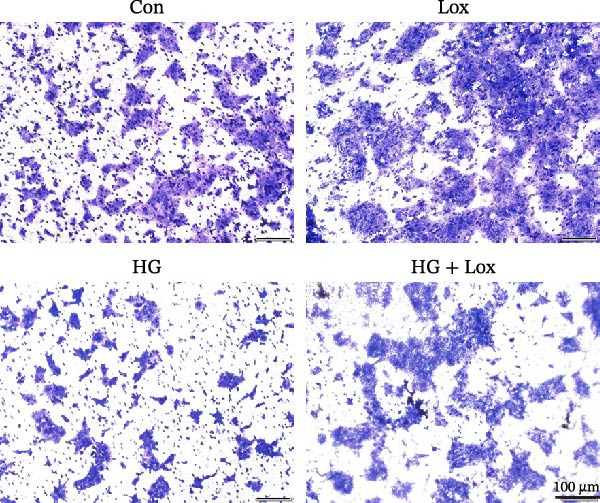
(C)

**Figure:**
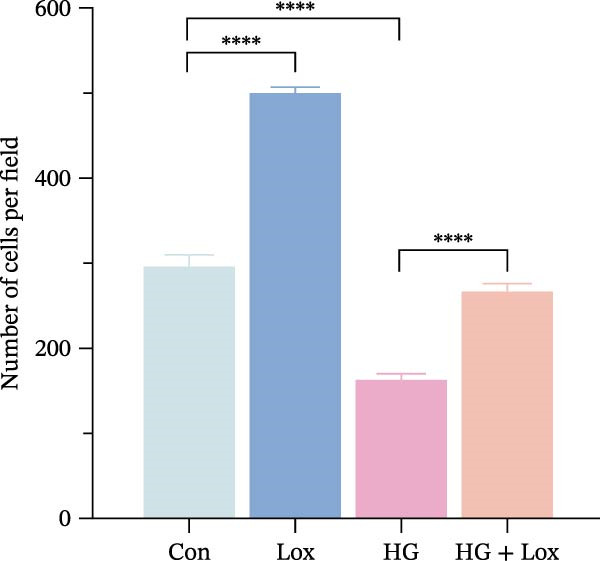
(D)

### 3.3. PEG‐Lox Activated PI3K/AKT/EGR1 Pathway in HaCaT Cells

Transcriptomic analysis was performed to evaluate gene expression changes in HaCaT cells following PEG‐Lox treatment. A Venn diagram revealed that 2970 genes were exclusively expressed in the PEG‐Lox‐treated cells (Figure 3A). A total of 288 DEGs were identified using the DESeq2 R package (1.20.0), comprising 198 up‐regulated genes and 90 down‐regulated genes (Figure 3B, C). The KEGG pathway analysis revealed that genes associated with cell proliferation, differentiation, apoptosis, and angiogenesis were significantly up‐regulated (Figure 3D). GO enrichment analysis revealed that top enriched terms among up‐regulated genes were associated with cell cycle G1/S phase transition, cell junctions, and cell adhesion (Figure 3E). Among the up‐regulated genes, EGR1, a zinc finger transcription factor that is closely related to cell proliferation and migration [26–31], was identified as a key DEG.

Figure 3PEG‐Lox activated PI3K/AKT/EGR1 pathway in HaCaT cells. (A) Venn diagram of overlapping and unique gene expression patterns across treatment conditions. (B) Heatmap visualization of mRNA expression profiles (|log2FC| >1, FDR <0.05) following PEG‐Lox treatment. (C) Volcano plot of differentially expressed mRNAs. (D) The top‐ranked signal pathways analyzed by the KEGG platform in PEG‐Lox treated group. (E) GO analysis of top enriched terms in biological processes (BP), cellular components (CC) and molecular functions (MF). (F) Western blotting analysis of PI3K, p‐PI3K, AKT, p‐AKT, EGR1. (G) Quantitative analysis of the protein levels of p‐PI3K/PI3K, p‐AKT/AKT and EGR1 (β‐actin was used as the internal control). Data are presented as mean ± SD from three independent experiments. Statistical differences are evaluated by two tail unpaired Student’s *t* test and one‐way or two‐way ANOVA.  ^∗^ *p* < 0.05,  ^∗∗^ *p* < 0.01,  ^∗∗∗^ *p* < 0.001,  ^∗∗∗∗^ *p* < 0.0001. Con, control; HG, high glucose; Lox, PEG‐Lox.

**Figure:**
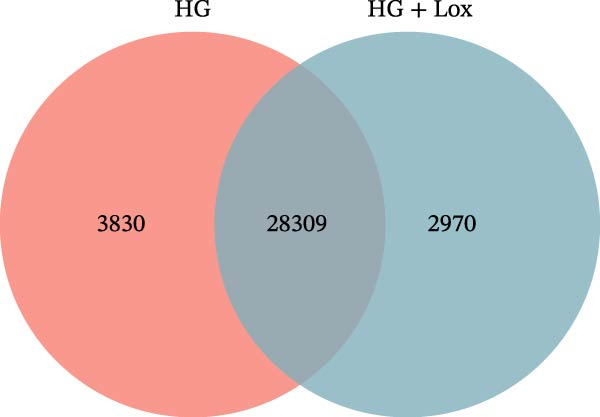
(A)

**Figure:**
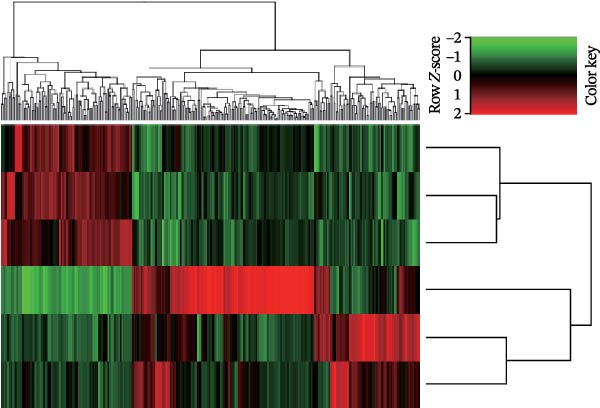
(B)

**Figure:**
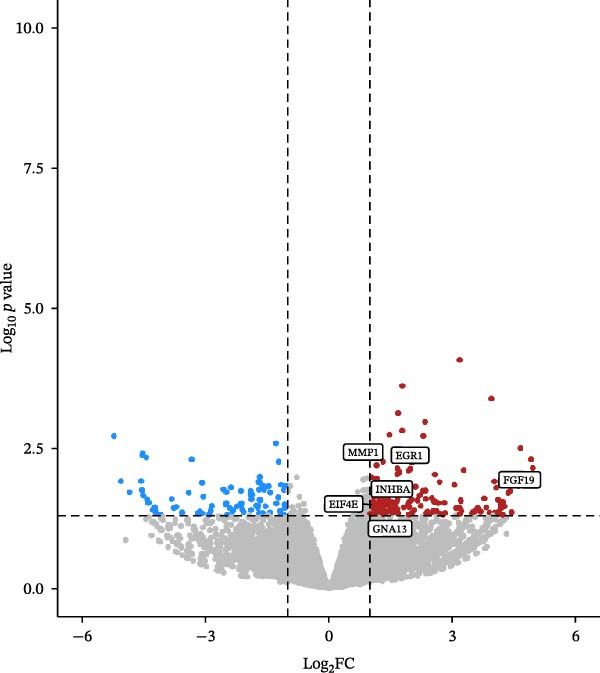
(C)

**Figure:**
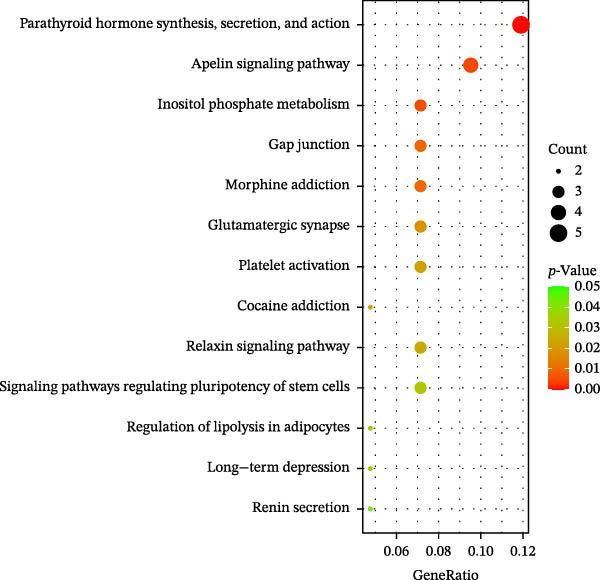
(D)

**Figure:**
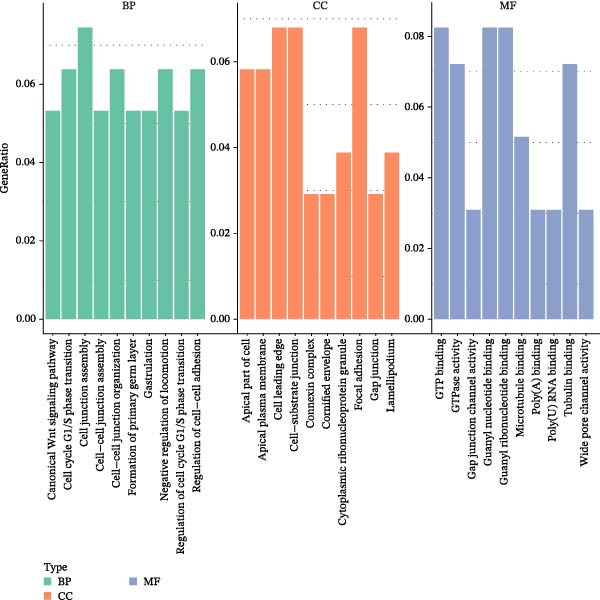
(E)

**Figure:**
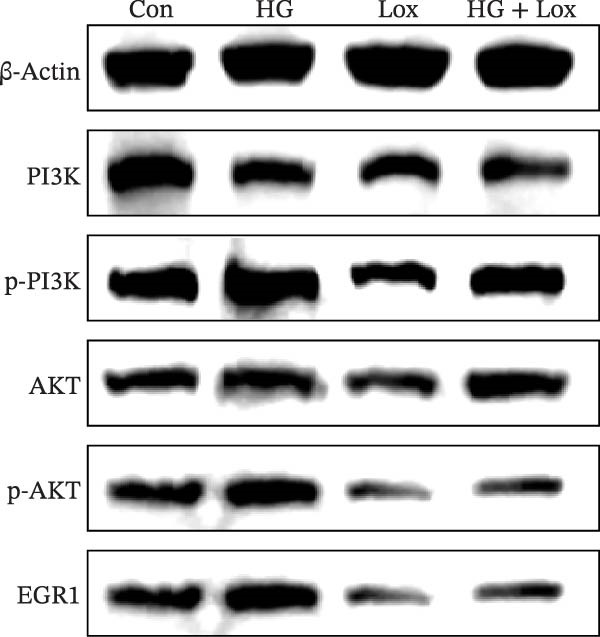
(F)

**Figure:**
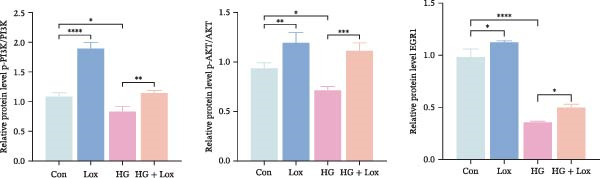
(G)

Previous studies have shown that EGR1 expression is regulated by the PI3K/AKT pathway [28, 32]. Subsequently, western blot analysis was performed to assess the expression of proteins related to the PI3K/AKT/EGR1 pathway (Figure 3F). The results demonstrated that HG downregulated the levels of p‐PI3K, p‐AKT and EGR1 (p‐PI3K/PI3K: Con group, 1.08 ± 0.07 vs. HG group, 0.83 ± 0.08, *p* < 0.05; p‐AKT/AKT: Con group, 0.94 ± 0.05 vs. HG group, 0.71 ± 0.04, *p* < 0.05; EGR1: Con group, 0.99 ± 0.07 vs. HG group, 0.36 ± 0.01, *p* < 0.0001). In contrast, PEG‐Lox treatment upregulated the expression of p‐AKT, p‐PI3K, and EGR1 under both NG and HG condition, corroborating our transcriptomic findings.

### 3.4. PEG‐Lox Induced Wound Healing In Vitro by Regulating PI3K/AKT/EGR1 Pathway

To further investigate the role of the PI3K/AKT/EGR1 pathway in the proliferation and migration of HaCaT cells induced by PEG‐Lox, HaCaT cells were treated with the PI3K inhibitor LY294002. Calcein AM/PI staining demonstrated that LY294002 significantly reduced the proportion of viable cells under the condition of PEG‐Lox (HG + Lox + LY group, 43.63 ± 3.3% vs. HG + Lox group, 70.13 ± 2.2%, *p* < 0.0001, Figure 4A, B). Similar findings were observed in the CCK‐8 assay (OD value: HG + Lox + LY group, 0.554 ± 0.021 vs. HG + Lox group, 0.990 ± 0.091, *p* < 0.001, Figure 4C). Furthermore, western blot analysis demonstrated that, despite PEG‐Lox supplementation, the expression levels of Cyclin D1 and Cyclin D3 were significantly decreased following LY294002 treatment (Cyclin D1: HG + Lox + LY group, 0.38 ± 0.03 vs. HG + Lox group, 0.87 ± 0.05, *p* < 0.0001; Cyclin D3: HG + Lox + LY group, 0.52 ± 0.07 vs. HG + Lox group, 0.86 ± 0.09, *p* < 0.01, Figure 4D, E). All these findings indicated that inhibition of PI3K abrogated the beneficial effect of PEG‐Lox on cell proliferation.

Figure 4LY294002 suppressed PEG‐Lox‐enhanced the biological functions of HaCaT cells. (A) Calcein AM/PI staining for the assessment of HaCaT cell viability. Green represents living cells; red represents dead cells. Scale bar: 100 µm. (B) Quantitative analysis of the proportion of living cells. (C) CCK8 assay for the cell proliferation evaluation. (D) Western blotting used to investigate the protein expression level of Cyclin D1 and Cyclin D3. (E) Quantitative analysis of the protein levels of Cyclin D1 and Cyclin D3 normalized to β‐actin. (F) Representative images of in vitro scratch wound healing assay at 12 h and 24 h. The black dotted line indicates the scratch area. Scale bar: 200 µm. (G) Quantitative analysis of the wound area (%) in vitro in the four groups. (H) Representative images of Transwell assay to evaluate cell migration. Scale bar: 100 µm. (I) Quantitative analysis of cell migration numbers of HaCaT cells. Data are presented as mean ± SD from three independent experiments. Statistical differences are evaluated by two tail unpaired Studentʼs *t* test and one‐way or two‐way ANOVA.  ^∗^ *p* < 0.05,  ^∗∗^ *p* < 0.01,  ^∗∗∗^ *p* < 0.001,  ^∗∗∗∗^ *p* < 0.0001. Con, control; HG, high glucose; Lox, PEG‐Lox; LY, LY294002.

**Figure:**
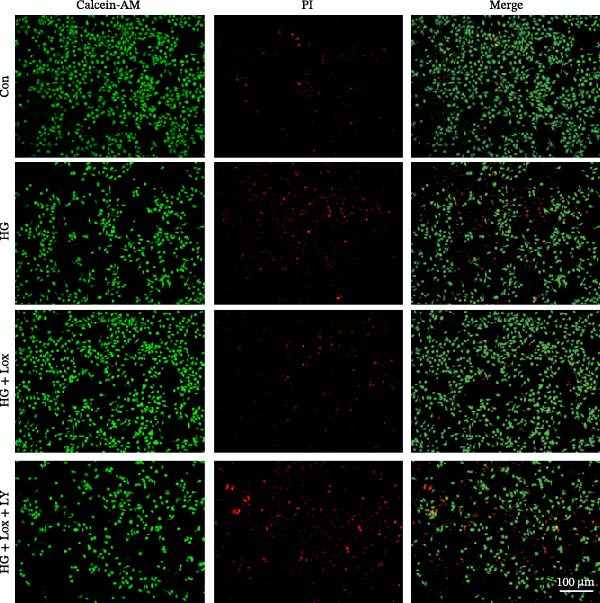
(A)

**Figure:**
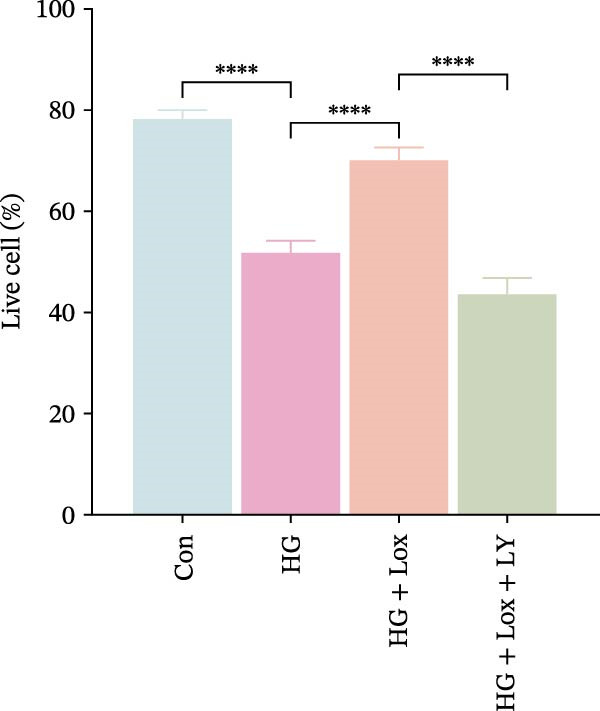
(B)

**Figure:**
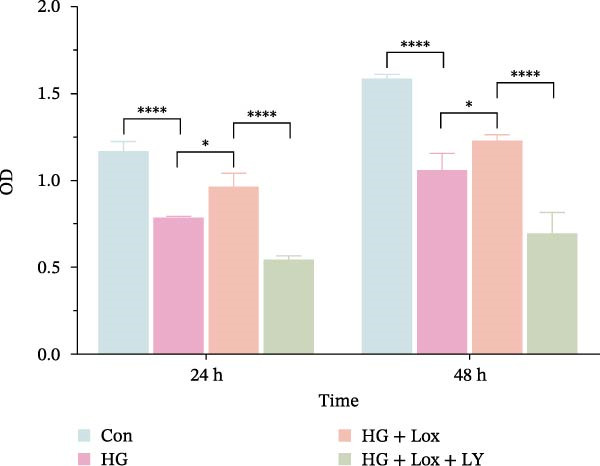
(C)

**Figure:**
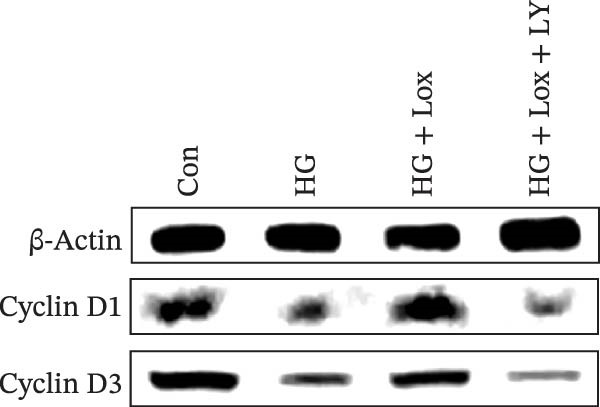
(D)

**Figure:**
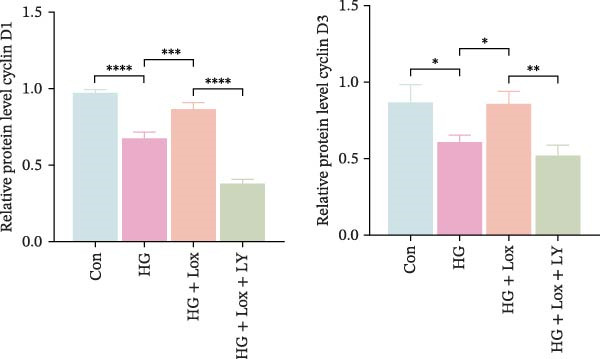
(E)

**Figure:**
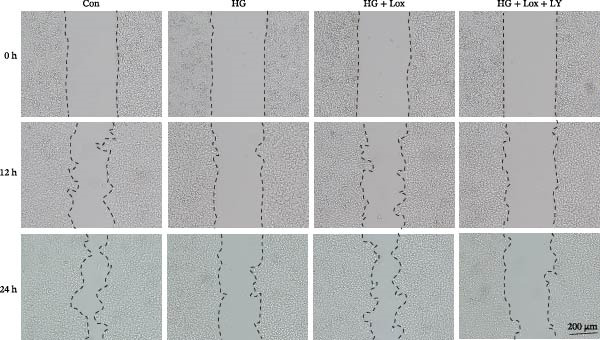
(F)

**Figure:**
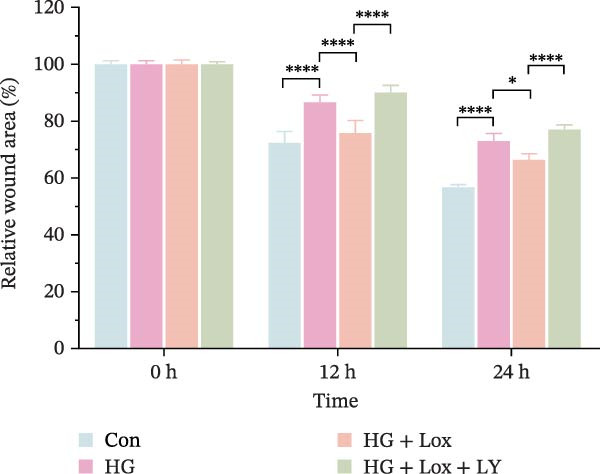
(G)

**Figure:**
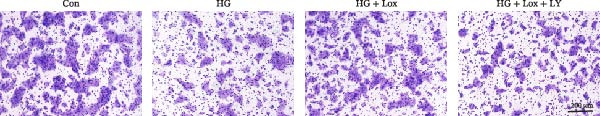
(H)

**Figure:**
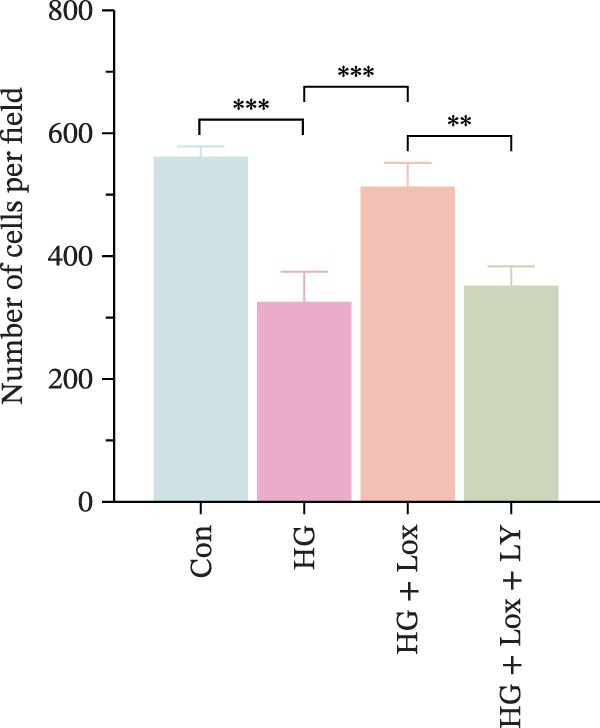
(I)

Consistently, scratch assay revealed that inhibition of the PI3K/AKT pathway markedly impaired cell migration induced by PEG‐Lox treatment (relative wound area: HG + Lox + LY group, 77.01% ± 1.66% vs. HG + Lox group, 66.38% ± 2.08%, *p* < 0.0001, Figure 4F, G). Comparable results were observed in the Transwell assay (Figure 4H, I).

Furthermore, western blot analysis demonstrated that, after LY294002 treatment, the expression levels of EGR1 were significantly decreased in PEG‐Lox‐treated cells (EGR1: HG + Lox + LY group, 0.46 ± 0.04 vs. HG + Lox group, 0.92 ± 0.10, *p* < 0.001, Figure 5A, D), verifying EGR1 as the essential downstream effector along the PI3K/AKT pathway in mediating PEG‐Lox’s pro‐wound healing effects.

Figure 5LY294002 suppressed the PEG‐Lox‐mediated activation of the PI3K/AKT/EGR1 pathway in HaCaT cells. (A) Western blotting used to investigate the protein expression of PI3K, p‐PI3K, AKT, p‐AKT, and EGR1. (B) Quantitative analysis of the protein level of p‐PI3K/PI3K. (C) Quantitative analysis of the protein level of p‐AKT/AKT. (D) Quantitative analysis of the protein level of EGR1 normalized to β‐actin. Data are presented as mean ± SD from three independent experiments. Statistical differences are evaluated by two tail unpaired Student’s *t* test and one‐way or two‐way ANOVA.  ^∗^ *p* < 0.05,  ^∗∗^ *p* < 0.01,  ^∗∗∗^ *p* < 0.001,  ^∗∗∗∗^ *p* < 0.0001. Con, control; HG, high glucose; Lox, PEG‐Lox; LY, LY294002.

**Figure:**
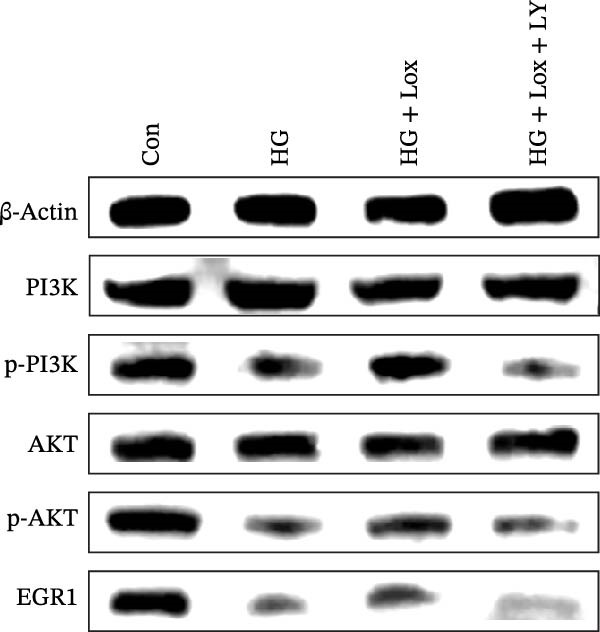
(A)

**Figure:**
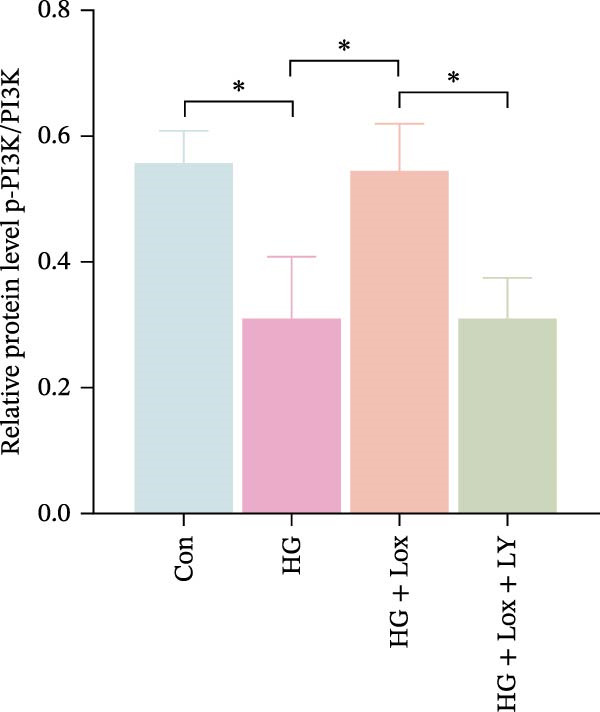
(B)

**Figure:**
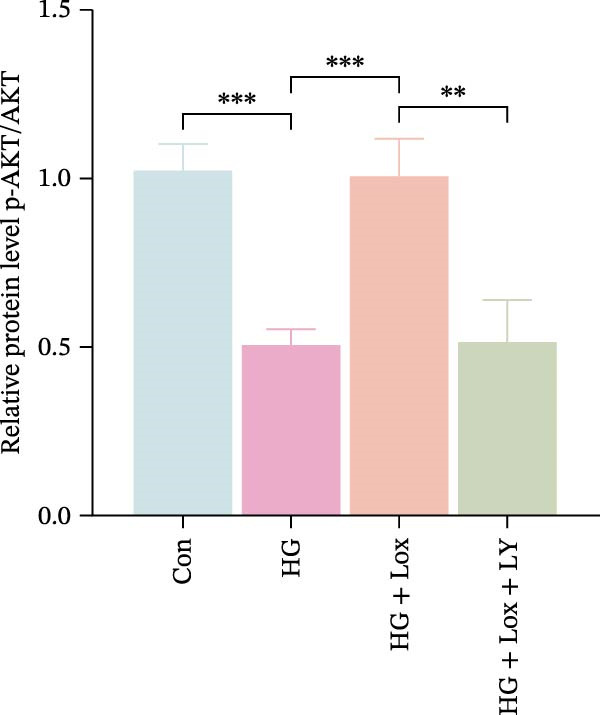
(C)

**Figure:**
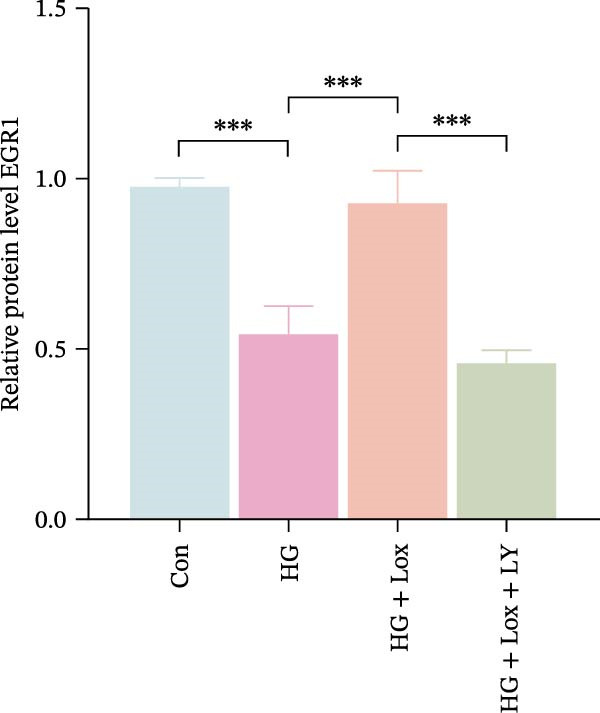
(D)

## 4. Discussion

Currently, research regarding the pro‐healing effects and underlying mechanisms of GLP‐1RAs in wound healing has primarily focused on daily‐administered agents like exenatide and liraglutide. Evidence on once‐weekly GLP‐1RAs remains critically limited. It is still unclear whether these extended‐release formulations exert similar impacts on wound repair. This study for the first time explored the ability of once‐weekly GLP‐1RA PEG‐Lox to improve wound healing and its underlying mechanisms in keratinocyte models.

It is well established that keratinocytes are essential for maintaining and restoring the skin barrier, as well as facilitating re‐epithelialization [13]. Hyperglycemia‐induced keratinocyte dysfunction significantly delays diabetic wound healing, yet its mechanisms remain poorly defined [15–17]. While the hyperglycemic milieu is known to impair keratinocyte differentiation and migration [16, 17], the broader mechanistic landscape is unclear. Building upon this foundation, our study further confirmed the specific deleterious effects of HG on the proliferation and migration of HaCaT cells in vitro, providing further insights into this critical pathological process.

Recent animal studies have investigated the effectiveness of GLP‐1RAs, specifically exenatide and liraglutide, in promoting wound healing in either normoglycemic rodents [5, 33] or diabetic rodents [18]. For example, our previous study demonstrated that liraglutide enhanced wound healing in normoglycemic mice with an optimal dosage of 2 µg/wound area (cm^2^) [7]. Mechanistically, GLP‐1RAs could facilitate wound healing by regulating the function of various wound cells [4–8]. However, only two in vitro studies have explored the effect of liraglutide on the function of HaCaT cells and the underlying mechanisms [5, 18]. In addition, the impact of ultra‐long‐acting GLP‐1RAs remains completely unknown. Here, our study demonstrated for the first time that the once‐weekly GLP‐1RA PEG‐Lox robustly improved HaCaT cell function, enhancing both proliferation and migration under NG condition. Furthermore, we presented the novel finding that PEG‐Lox conferred effective protection against glucotoxicity‐induced impairment in HaCaT cells. This finding is of particular significance, as PEG‐Lox not only improved basal keratinocyte function but also mitigated diabetes‐associated dysfunction, thereby underscoring its potential therapeutic relevance for diabetic and non‐diabetic wound healing. Critically, the pro‐proliferative effect of PEG‐Lox was sustained for at least 48 h. This key finding provides a mechanistic rationale and early evidence supporting the potential for an extended dosing interval in vivo, which warrants further investigation in preclinical models.

Recent studies have emphasized the importance of the PI3K/AKT pathway in keratinocyte proliferation and migration [34, 35]. Our data identified that PEG‐Lox could regulate HaCaT proliferation via the PI3K/AKT pathway in HaCaT cells, consistent with the findings from Nagae et al. [5]. However, the downstream effectors of this pathway were not identified in their study. To address this gap, we investigated the downstream signaling events via RNA sequencing and bioinformatics analysis. We identified that the expression of EGR1 was significantly up‐regulated after PEG‐Lox treatment, which was further confirmed by western blot analysis. EGR1 is a zinc finger transcription factor that plays an essential role in the proliferation and migration of various cells, including vascular endothelial cells [26], immune cells [27], keratinocytes [29], and tumor cells [31]. Critically, EGR1 has been established as a key mediator in HaCaT cell proliferation, functioning as a key downstream effector in the epidermal growth factor (EGF) mitogenic pathway [29]. In addition, EGR1 can directly regulate the expression of cell cycle‐related proteins [30, 36]. Therefore, we speculated that EGR1 may act as a key effector for PEG‐Lox in modulating the function of keratinocytes.

Previous studies have established that the PI3K/AKT pathway directly regulates the EGR1 transcription across diverse cell types. For instance, this pathway has been shown to mediate EGR1‐dependent proliferation of smooth muscle cells induced by low density lipoprotein (LDL) [28] and to be indispensable for proliferative signaling in Jurkat cells [32]. However, whether the PI3K/AKT signaling pathway regulates EGR1 in keratinocytes has not been elucidated. Based on our experimental data, pharmacological inhibition of PI3K using LY294002 effectively suppressed both PEG‐Lox‐induced AKT phosphorylation and subsequent EGR1 expression in HaCaT cells. These results clearly demonstrate that the PI3K/AKT pathway serves as the essential upstream regulator mediating PEG‐Lox‐induced EGR1 expression. Our findings reveal a previously unrecognized signaling axis, PI3K/AKT/EGR1 pathway, through which PEG‐Lox enhances HaCaT cell proliferation and migration. This newly identified pathway not only deepens our understanding of the molecular mechanisms underlying PEG‐Lox’s bioactivity but also provides a novel mechanistic foundation for its therapeutic potential in wound healing.

Furthermore, our findings hold significant translational value, particularly highlighting the therapeutic advantage of ultra‐long‐acting GLP‐1RA analogs such as PEG‐Lox. Unlike short‐acting agents, which require frequent administration during wound healing process, the prolonged pharmacokinetic profile of PEG‐Lox supports sustained receptor activation and stable pro‐healing effects. This inherent ultra‐long‐acting nature has the potential to reduce dosing frequency and ensure continuous exposure to the therapeutic agent, which is critically aligned with the extended healing process characteristic of diabetic foot ulcers (DFUs). Consequently, our findings on PEG‐Lox represent a pharmacologic improvement, offering a clinically meaningful advancement over short‐acting analogs and providing a more practical and effective treatment paradigm for chronic wound management. Moreover, as the effects of PEG‐Lox on keratinocytes were evident irrespective of glucose levels, its therapeutic potential could also extend to other complex non‐diabetic wounds, such as pressure ulcers and traumatic wounds. Most importantly, by identifying EGR1 as a central mechanistic mediator, our work not only provides a rationale for repurposing PEG‐Lox but also uncovers a novel therapeutic target for future pharmacological intervention in wound healing. Strategies aimed at stabilizing or potentiating EGR1 activity may thus represent a promising new avenue for therapeutic development.

There are some limitations in this study. First, it was performed in HaCaT cells, lacking the complex biological interactions found in vivo. It is difficult to represent the various types of cells and pathological characteristics of the wound conditions with simple HaCaT cell models in vitro. However, our in vitro findings provided a clear, testable hypothesis for further in vivo validation. Studies that investigate the pro‐healing effects of PEG‐Lox in animal models and patients with diabetic ulcers are ongoing. In addition, we also titrated different dosing intervals to demonstrate the advantages of ultra‐long‐acting GLP‐1RAs over daily GLP‐1RAs on wound healing. Second, in vivo experiments are necessary to directly assess the role of EGR1 in the different stages of wound healing.

## 5. Conclusion

Our study provides the first evidence that the ultra‐long‐acting GLP‐1RA PEG‐Lox potently activates keratinocyte function via the PI3K/AKT/EGR1 pathway, revealing a previously unrecognized mechanism of action. These findings broaden the therapeutic scope of GLP‐1RAs beyond their traditional role in metabolic regulation, and position PEG‐Lox as a promising candidate for the treatment of diabetic wounds and other types of chronic wounds with longer dosing intervals.

**Nomenclature**
AKT: Serine/threonine kinase
AMPK: AMP‐activated kinase
BMI: Body mass index
CCK8: Cell counting kit 8
CD34/KDR: Circulating endothelial progenitor cells
DFUs: Diabetic foot ulcers
DGE: Differentially expressed genes
DMEM: Dulbecco’s modified Eagle’s medium
EGF: Epidermal growth factor
EGR‐1: Growth response factor‐1
GLP‐1Ras: Glucagon‐like peptide‐1 receptor agonists
GLP‐1: Glucagon‐like peptide‐1
GLP‐1R: Glucagon‐like peptide‐1 receptor
GO: Gene ontology
HaCaT cells: Human skin keratinocytes
HG: High glucose
HUVECs: Human umbilical vein endothelial cells
KEGG: Kyoto encyclopedia of genes and genomes
LDL: Low density lipoprotein
MMP‐9: Matrix metalloproteinase‐9
NG: Normal glucose
OD: Optical density
PEG‐Lox: Polyethylene glycol loxenatide
PEG: Polyethylene glycol
PI: Propidium iodide
PI3K: Phosphatidylinositol 3 kinase (PI3K)
PKC: Protein kinase C
p‐PI3K: Phosphorylated PI3K
p‐AKT: Phosphorylated AKT
TIMP‐1: Tissue inhibitor of metalloproteinase‐1
T2DM: Type 2 diabetes mellitus
SDS‐PAGE: Sodium dodecyl sulfate‐polyacrylamide gel electrophoresis
PVDF: Subsequently transferred to a polyvinylidene fluoride.

## Author Contributions

Methodology: Shiying Shao and Zhiyi Zhao. Software: Han Yue. Formal analysis and investigation: Zhiyi Zhao, Weijie Xu, and Song Gong. Writing – Original Draft Preparation: Zhiyi Zhao. Writing – Review & Editing: Shiying Shao and Song Gong.

## Funding

This work was supported by a grant from Hubei Provincial Natural Science Foundation and YICHANG‐of China (Grant number 2025AFD271 to Shiying Shao), a grant from China International Medical Foundation‐Senmei China Diabetes Research Fund (Grant number Z‐2017‐26‐1902‐5 to Shiying Shao), a grant from the fund of Sichuan Provincial Western Psychiatric Association’s CSPC LEADING Scientific Research Project (Grant number WL2021104 to Shiying Shao), and a grant from Bethune Charitable Foundation (Grant number 2024 to Shiying Shao).

## Ethics Statement

The HaCaT cell line used in this study is an established immortalized cell line, obtained through legitimate commercial channels from Servicebio (Wuhan, China) (Catalog No.: STCC11801). This article is based on previously conducted studies and does not contain any new studies with human participants or animals performed by any of the authors.

## Conflicts of Interest

The authors declare no conflicts of interest.

## Data Availability

The raw data supporting the conclusions of this article will be made available by the authors upon request.
